# Twenty‐five years of addressing cutting‐edge scientific, policy, and regulatory issues through collaboration: The Forum for Collaborative Research

**DOI:** 10.1111/cts.70051

**Published:** 2024-10-19

**Authors:** Robin Schaefer, Alessi Ayvaz, Christopher R. Hoffman, Margot Yann, Zachary Rooney, Mitchell Leus, Shilpa Mitra, Veronica Miller, for the Forum for Collaborative Research

**Affiliations:** ^1^ Forum for Collaborative Research University of California, Berkeley Washington DC USA

## Abstract

Developing safe and effective drugs and other medical products is a complex and costly process. Drug development has been, historically, commonly competitive and uncollaborative, and this tendency toward a lack of interaction between stakeholders—the pharmaceutical industry, academia, regulatory agencies, healthcare providers, and communities, among others—can lead to missed opportunities to improve efficiency and, ultimately, public health. The Forum for Collaborative Research was established in 1997 to address current scientific, policy, and regulatory issues in global health through multistakeholder engagement and dialogue. By providing a neutral and safe space for discussion, the Forum's model has impacted how clinical trials in diverse health areas are conducted, supported broader and more equitable clinical trial participation, and accelerated delivery of new drugs. The Forum's focus and directions have shifted over time, and this responsiveness to the needs of the global health community will be critical to ensure that the Forum continues to support collaboration in global health. In this article, we present lessons learned from this innovative model of collaborative research and regulatory science, pioneered by the Forum for over 25 years, including the importance of collective ownership and governance by all stakeholders, and emphasis on common goals and advantages of collaboration.

## INTRODUCTION

Developing safe and effective drugs and other medical products—and ensuring access for those in need—is a multifaceted, iterative process, involving a large number of stakeholders. Costs of drug development have been increasing significantly over time.^1^ Clinical trials required for drug approval are increasingly complex and globalized. In some areas of health, traditional placebo‐controlled study designs may no longer be feasible or ethical, requiring innovative study designs. Biomarkers are increasingly used in drug development and have the potential to increase the efficiency of clinical trials,^2^ particularly when they serve as surrogate end points, requiring consensus between sponsors of clinical trials and regulatory authorities on the most appropriate biomarkers. Despite the increasing need for and benefit of collaboration, drug development has been, historically, commonly a competitive and uncollaborative process, with individual companies engaging with specific regulatory agencies and academic partners as it concerns the development of a drug. The involvement of patient and community groups is often limited. The tendency toward a lack of interaction between stakeholders—the pharmaceutical industry, academia, regulatory agencies, healthcare providers, and communities, among others—can lead to missed opportunities to improve the efficiency of drug development and, ultimately, public health.

The Forum for Collaborative Research (henceforth the Forum) was established in 1997 against this backdrop of the need for multistakeholder collaboration in global drug development. It is part of the University of California, Berkeley, School of Public Health, with offices in Washington, DC. It aims to address current scientific, policy, and regulatory issues in global health through multistakeholder engagement and dialogue. The original impetus for the establishment of the Forum was the development of new antiretrovirals for the treatment of HIV/AIDS, which required increasingly complex and collaborative research. The Forum provided a platform for pharmaceutical companies, academic researchers, government agencies, funding organizations, and community groups to collaboratively identify gaps in HIV research and the necessary steps to address these gaps. Since then, the Forum has addressed critical issues in diverse health areas, including hepatitis B virus and C virus (HBV and HCV), liver diseases, primary sclerosing cholangitis (PSC), transplantation‐associated virus infections (TAVI), retinal ocular diseases, rare genetic diseases, and COVID‐19. In this article, we present lessons learned from this innovative model of collaborative research and regulatory science, pioneered by the Forum for over 25 years.

## THE FORUM'S APPROACH: THEORETICAL FOUNDATIONS

**FIGURE 1:**
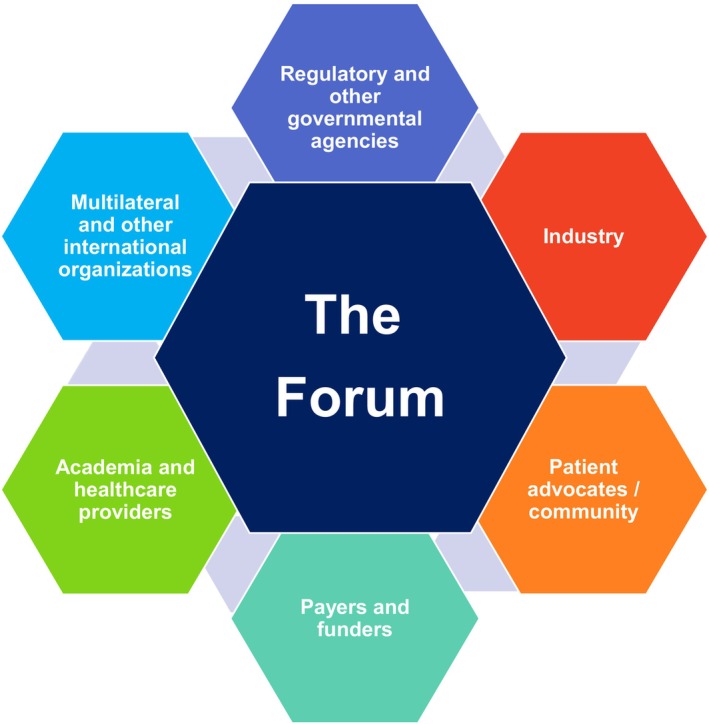
The approach of the Forum for Collaborative Research (the Forum) of creating, maintaining, and directing multistakeholder networks, as relevant to the specific disease area and project. These networks are governed and owned collectively by their participants. At the same time, the Forum acts as the Network Administrative Organization that sustains and coordinates the network while allowing for inclusive and democratic decision‐making.

The Forum aims to address health‐related scientific, policy, and regulatory issues by engaging diverse stakeholders and fostering dialogue and consensus. It operates through disease‐specific forums (Table 1), which, as of 2024, include the HIV Forum, HBV Forum, Liver Forum, PSC Forum, TAVI Forum, and Ocular Diseases Forum. These forums include invited participants from academia, regulatory and other governmental agencies, multilateral and other international organizations, community organizations (such as patient advocacy groups), healthcare providers, payers and funders, and industry, as is relevant (Figure 1). It is, therefore, important to distinguish between the Forum for Collaborative Research as an organization (“the Forum”) and the disease‐specific forums as the networks it creates and facilitates. Each forum has a steering committee and consists of working groups addressing specific areas of interest. The steering committee comprises key opinion leaders from their respective stakeholder groups and provides overall scientific guidance, leadership, and expertise. While all stakeholders can raise topics for consideration by the Forum, the steering committee's guidance is critical for identifying which issues are addressed through working groups.

**TABLE 1:** Active programs and projects of the Forum for Collaborative Research (as of 2024).

Program/project	Disease(s) addressed	Year established	Example outputs
HIV Forum	HIV	1997	See references^10^, ^11^, ^12^, ^13^
TAVI Forum^a^	CMV, BK virus, AdV, HHV 6, HSV, measles, respiratory viruses (RSV, influenza)	2014	See references^20^, ^21^, ^22^
Liver Forum	MASH, MASLD	2014	See references^23^, ^24^, ^25^
HBV Forum	HBV, HDV	2016	See references^15^, ^16^, ^17^, ^18^, ^19^
PSC Forum^b^	PSC	2017	PSC Forum meetings 1–7 (2017–2024)^c^
PCLD program^d^	Pediatric PSC, Alagille syndrome, biliary atresia, progressive familial intrahepatic cholestasis	2021	PCLD Workshop 1, Milan, Italy, May 14, 2024^e^
Data and Analysis Center	A neutral venue for data sharing and analysis across the Forum's project areas	2022	MASH Placebo Database Project^f^
Ocular Diseases Forum	Inherited retinal diseases, age‐related macular degeneration	2023	Ocular Diseases Forum 1, Los Angeles, CA, USA, May 15, 2023^g^
COVID‐19 project	COVID‐19	2023	COVID‐19 Therapeutics Development Workshop, Washington, DC, USA, November 2, 2023

*Note*: Further information, resources, and peer‐reviewed publications across programs/projects can be found online: https://forumresearch.org/.

Abbreviations: AdV, adenovirus; CMV, cytomegalovirus; HBV, hepatitis B virus; HCV, hepatitis C virus; HDV, hepatitis D virus; HHV 6, human herpes virus 6; HIV, human immunodeficiency virus; HSV, herpes simplex virus; MASH, metabolic dysfunction‐associated steatohepatitis; MASLD, metabolic dysfunction‐associated steatotic liver disease; PCLD, pediatric cholestatic liver diseases; PSC, primary sclerosing cholangitis; RSV, respiratory syncytial virus; TAVI, transplantation‐associated virus infection.

^a^ Formerly known as the Cytomegalovirus Forum (CMV Forum).

^b^ The PSC Forum is part of the Liver Forum.

^c^ Materials from PSC Forum meetings are available online: https://forumresearch.org/psc‐forum/psc‐forum‐meetings.

^d^ The PCLD program is part of the Liver Forum and is not a formal forum.

^e^ Materials from the PCLD Workshop are available online: https://forumresearch.org/liver‐forum/pediatric‐cholestatic‐disease‐program.

^f^ Further information on the MASH Placebo Database Project is available online: https://forumresearch.org/data‐center/projects/nash‐placebo‐arm‐database.

^g^ Materials from the Ocular Diseases Forum are available online: https://forumresearch.org/ocular/meetingsoculardisease/1803‐ocular‐diseases‐forum‐1.

The Forum, therefore, comprises networks (disease‐specific forums), which, in turn, comprise sub‐networks (steering committees and working groups). These networks comprise autonomous organizations and individuals connected through a common goal. As such, the Forum has characteristics of what has been referred to as an “ecosystem orchestrator”^3^ or “hub firm.”^4^ These terms, which originated in business management research, describe how an actor can design and shape networks despite lacking formal authority. However, the Forum emphasizes that it is collectively owned by its stakeholders and democratically governed by its members. Therefore, the networks established by the Forum are both governed collectively by their participants and governed by the Forum as the “Network Administrative Organization,” as described by Provan and Kenis in their seminal work on network governance.^5^ The Network Administrative Organization sustains and coordinates the network while allowing for inclusive and democratic decision‐making. In their work on public administration governance, Emerson and colleagues described this as “collective governance,”^6^ which is the collective orchestration of collective action. In their framework, the Forum's role can be seen as that of a leader who initiates a collective governance regime without becoming the leader of the collaboration. To achieve collective governance, steering committees and working groups aim for consensus in decision‐making. However, complete consensus—acceptance of decisions by all members—may not always be achieved. In these situations, decisions are made based on the principle of consent, similar to sociocratic organizations. These are the decisions to which no group member has any fundamental and constructive objection.

The members of the networks convened by the Forum are motivated by the belief that a goal could not be achieved by any participant in the network alone or that the goal could be achieved more efficiently and effectively through collaboration (the “collaborative advantage”^7^). This is partly driven by the growing complexity of drug development and global health. A key task of the Forum is to establish and maintain trust between participants in collaborative networks.^8^ To achieve this, the Forum emphasizes the common goal of addressing a public health need, so the outcomes of the Forum's activities are public goods and do not just benefit network participants. Moreover, the Forum facilitates “knowledge mobility” and manages “knowledge appropriability.”^4^ Knowledge exchange between network members is facilitated by creating a “safe space” for deliberations and discussions. The emphasis on creating a public benefit ensures that the value created by the network is distributed equally among members. Democratic decision‐making and emphasis on consensus further foster a sense of joint ownership of the value generated by collaborative actions.

The Forum is an independent body that has no direct financial interest in the activities of the networks it convenes. The coordination by such a “neutral convener” has been found to be an important factor for the success of consortia, for instance, in the pharmaceutical^9^ and nanoelectronics industry.^3^ The Forum is funded through research grants and contributions from its stakeholders. Participation of industry partners in the Forum's activities is conditional on non‐disclosed annual sponsorship contributions, depending on the company's size. Contributions from non‐industry organizations are encouraged. While sponsorship includes participation in the Forum's activities, participating members of industry partners must be scientific or regulatory experts engaged in the relevant field they participate in. The Forum's meetings are not venues for marketing and investment experts. This funding model contributes to the sense of collective ownership and prevents individual stakeholders from having outsized influence on the work.

Collaborations in networks facilitated by the Forum commonly go through an iterative process. Collaborations start with at least some shared motivation, highlighted by the Forum's emphasis on a common goal. The Forum facilitates initial engagement between network members, which can strengthen the sense of shared interest and purpose. This, in turn, increases trust between network members, leading to more engagement and creating a “virtuous cycle” of collaborative dynamics.^6^ Network participants commonly have long‐standing relationships with the Forum and other network members, which supports the trust and engagement in networks.

## CHANGING FOCUS, EXPANDING SCOPE

The first applications of the Forum's model involved diverse stakeholder convenings addressing issues in HIV treatment. This focused on building consensus around clinical trial designs,^10^ including definitions of patient populations and non‐inferiority study designs, as well as pharmacovigilance.^11^ More recently, the HIV Forum's focus shifted toward innovations in HIV prevention. For instance, in 2011, the Forum provided a neutral space to discuss implementation of pre‐exposure prophylaxis (PrEP) in the US.^12^ With an increasing number of PrEP options becoming the standard of preventative care, placebo‐controlled trials are no longer considered ethical, and non‐inferiority or superiority trials may not be feasible due to the need for unpractically large sample sizes. The Forum's convening facilitated consensus around innovations in HIV prevention clinical trials using background HIV incidence as a comparator,^13^ which has critically informed the design of currently ongoing PrEP clinical trials. The belief in a common goal (establishing acceptable trial designs) between diverse stakeholders (industry, academia, regulatory agencies, and organizations representing communities affected by HIV) was essential in achieving this consensus. Similarly, the Forum could build on long‐standing relationships, due to its history of facilitating consensus around trial design in HIV treatment, which supported engagement and trust between stakeholders.

While HIV has remained a key part of the Forum's work throughout its history, the Forum expanded significantly in scope. In 2007, after successes in support of HIV treatment development, companies developing new antiviral drugs for HCV infection requested the Forum's support in standardizing drug resistance information for regulatory review. This led to the HCV Drug Development Advisory Group, which expanded its scope to address other issues in HCV clinical trials. An evaluation of the work of this group found that the key to its success was its informal, science‐driven tone during meetings and the diversity and comprehensiveness of its membership, including representation from academia, industry, regulatory agencies, and patient communities.^14^ The funding model, which involved non‐disclosed contributions by pharmaceutical and diagnostic companies, was similarly highlighted as contributing to the group's success as it fostered a sense of joint ownership, with the project benefiting the field as a whole and not an individual company or product. The expansion of the Forum's scope to HCV also highlights a key characteristic of the Forum: its responsiveness to the priorities and needs of its stakeholders, ensuring that it addresses the most pressing and relevant issues.

Given successes in developing HCV treatment and cure, the HCV Forum steering committee and other stakeholders encouraged to shift attention to facilitating the development of similarly safe and effective therapies for HBV. Major accomplishments of the HBV Forum include identifying needs and gaps in HBV drug development,^15^ proposing considerations for designing clinical trials of novel combination therapies,^16^ strengthening the evidence base around surrogates and biomarkers,^17^ and developing consensus on how to stop novel therapies in clinical trials to assess safety and efficacy.^18^ Given that coinfection of HBV and hepatitis Delta virus (HDV) leads to the most severe form of chronic viral hepatitis, the HBV Forum expanded its scope to address issues in HDV drug development. This work has led to a summary of available HDV RNA assays to quantify performance variability, advocating for the development of standardized, reliable, and accurate HDV RNA assays for antiviral treatment response monitoring and diagnosis.^19^

In 2014, the Forum's model of facilitating informal but structured interactions between diverse stakeholders who collectively own the projects and drive progress through consensus was extended to TAVI and liver diseases as the first non‐communicable disease challenges. The TAVI Forum began as the Cytomegalovirus (CMV) Forum and aimed to address treatment and prevention options for CMV in transplant recipients. In solid‐organ and hematopoietic stem‐cell transplant recipients, latent viral infections (such as with CMV) can be reactivated, causing life‐threatening infections after transplantation. CMV Forum stakeholders have developed standardized disease definitions and recommendations for clinical trial designs,^20^ including surrogate markers for disease outcomes.^21^ The success of the CMV Forum inspired discourse on additional transplant‐associated viruses, such as BK virus, Adenovirus, and Human Herpes Virus 6. To encompass these additional viruses, the CMV Forum became the TAVI Forum in 2019, which most recently published consensus on outcome definitions for BK virus trials.^22^ The developments in the TAVI Forum underscore, again, how collaboration can lead to a virtuous cycle in which trust between network participants increases through successful collaboration (in the area of CMV), leading to interest in collaborating further on related issues (such as BK virus).

The Liver Forum focuses on metabolic dysfunction‐associated steatotic liver disease (MASLD), including metabolic‐associated steatohepatitis (MASH), a leading cause of chronic liver disease‐associated morbidity and mortality. Chronic conditions such as MASLD have a long asymptomatic natural history. This creates challenges for clinical trials on therapeutics as clinical end points, such as cirrhosis or death, require unfeasibly long follow‐up times. To address these challenges, the Forum, acting as the Network Administrative Organization, established and coordinates diverse networks including representation from academia, regulatory agencies, industry, and patient organizations. Multistakeholder dialogue facilitated by the Liver Forum generated recommendations on case definitions in clinical trials,^23^ data harmonization and standardization,^24^ and clinical trial end points.^25^ As with other forums, the agenda of the Liver Forum is informed by its members, with a steering committee identifying priorities addressed by working groups. Successful consensus building in the Liver Forum has led to the establishment of a program on pediatric cholestatic liver disease (PCLD) and the PSC Forum.

Most recently, the Forum's model was applied to build consensus around the identification and validation of outcome measures in clinical trials for ocular diseases, specifically inherited retinal diseases (IRDs) and age‐related macular degeneration. The Ocular Disease Forum was founded in 2021, after the Forum was approached by a member of the Liver Forum, noting huge gaps in the retinal disease research landscape. The Ocular Disease Forum highlights how the Forum continues to be responsive to public health needs identified by the research community. Similarly, in response to the COVID‐19 pandemic and the continuing need to develop effective therapeutics in light of ever‐changing virus variants, the Forum has initiated a series of meetings to establish acceptable trial designs for COVID‐19 therapeutics. This builds on long‐standing relationships of the Forum with stakeholders involved in the development of antiviral drugs and the trust these stakeholders have in the Forum as a neutral convener. The first public meeting, held at the end of 2023, which included participation from regulatory agencies, focused on the ethics and feasibility of different trial designs, patient eligibility criteria, suitable control groups, and clinical and surrogate virologic end points. The meeting highlighted solutions for improving risk assessment and treatment strategies, focusing on optimizing patient outcomes while ensuring scientific rigor and ethical conduct. There was extensive discussion around medically attended visits (MAVs) as a primary efficacy end point in trials for patients with mild‐to‐moderate COVID‐19. However, multiple stakeholders expressed skepticism on whether MAVs would serve as a clinically meaningful end point, advocating instead for a focus on symptom‐based end points and early intervention strategies. In future meetings, stakeholders will continue to deliberate on the most appropriate end points and trial designs, highlighting how the Forum's approach commonly involves an iterative process to build consensus. Future meetings will also aim to address Long COVID‐19 and post‐COVID‐19 conditions.

## NEW STRATEGIC DIRECTIONS

While the Forum's scope and focus have shifted over time, the core principles of its model—providing a safe space for multistakeholder dialogue to advance public health through consensus building—have remained constant. In 2022, the Forum established the Data & Analysis Center (DAC), representing both a new strategic direction and an evolution of the Forum's approach. While it has been common across the Forum's areas that stakeholders identify research questions to support their own analyses, the DAC will allow the Forum to aggregate data and perform analyses using standard and innovative analytical techniques to benefit all stakeholders involved.

The DAC emerged from extensive discussions among the Forum's stakeholders and the identified need for and benefit of a neutral space for data analyses that could provide broad benefits to all stakeholders. The DAC is designed to provide an innovative data science platform for aggregating and analyzing individual‐level data in the areas that the Forum's networks have decided to focus on. The DAC, and the data sharing it requires, is based on the trust between stakeholders built by the Forum through its approach of collective governance, facilitating willingness by pharmaceutical companies and other partners to share data. Research on health data sharing has identified that academic institutions are viewed as more trustworthy stewards of data than other types of organizations,^26^ further differentiating the DAC—housed within the University of California, Berkeley, School of Public Health—as a neutral space for data sharing. Furthermore, the Forum's history of working at the intersection of drug development and regulatory processes increases the likelihood that the outcomes of DAC projects will lead to meaningful public health impact.

The DAC is a comprehensive platform for securing data to meet the requirements of the Forum's stakeholders. The DAC platform is housed in a secure enclave platform built for University of California, Berkeley, researchers and is approved by the campus for highly sensitive data. As such, the platform is aligned with the administrative, policy, and procedural controls required by regulations and policies such as the US Health Insurance Portability and Accountability Act (HIPAA) and the European Union General Data Protection Regulation (GDPR).

In line with the Forum's model, analyses conducted by the DAC will be directed by the Forum's stakeholders who will form working groups to identify research questions and develop statistical analysis plans. The DAC will have sole access to the data and implement the analyses. An exception to this is where analyses aim to support regulatory processes, and the data use agreements developed by the Forum allow for regulatory authorities to access the data. The first project of the DAC is the Liver Forum's Placebo Database (PDB), an integrated patient‐level database of completed phase II and phase III clinical trials on therapeutics for MASH. The DAC will analyze individual‐level data of patients in the placebo cohorts of these trials. The analyses aim to support the Liver Forum's efforts to investigate the natural history, progression, and correlates of liver‐related fibrosis and potential clinical events of MASH, and to identify key biomarkers for MASH fibrosis and progression. Outcomes of this study can inform strategic patient selection in clinical trials for MASH.

## THE FUTURE OF THE FORUM'S MODEL

By providing a safe space for discussion of pressing scientific and regulatory issues, the Forum's model has impacted how clinical trials in diverse health areas are conducted, supported broader and more equitable clinical trial participation, and accelerated delivery of new drugs. Collective ownership and governance, with the Forum as the Network Administrative Organization, has been central to the Forum's impact and ensured that the Forum is addressing critical issues, as identified by the community that constitutes the networks it facilitates. Challenges to drug development, including complexity and costs, are likely to increase further, so the need to unlock the collaborative advantage is more pressing than ever. Most new drugs will be competing with existing ones rather than addressing a previously unmet need. Efficiency in drug development could be improved through the rise in artificial intelligence (AI) and machine learning, particularly in combination with real‐world data (RWD). Challenges with potential biases and reproducibility of results highlight the need for consensus on the ethical use of AI in drug development, and positive examples and clearer regulatory guidance are emerging.^27^ A workshop on AI in drug development by the US Food and Drug Administration in August 2024 emphasized the need for robust collaboration between industry and other stakeholders.^28^ Through its DAC, the Forum is already expanding its multistakeholder work in this area, and it is the Forum's ability to unite industry, academic, patient, and regulatory bodies that will provide a strong foundation for these efforts. Importantly, these efforts will be grounded in the Forum's focus on developing approaches that help sponsors, and the healthcare community at large, meet the evidentiary requirements of regulators.

The Forum is part of a broader universe of efforts and organizations that aim to bring diverse stakeholders together to foster collaboration and identify shared problems and opportunities in drug development and global health. These may focus on specific diseases (e.g., the Alzheimer's Disease Neuroimaging Initiative^29^) or may have a broader scope (e.g., the Accelerating Medicines Partnership^30^). A key characteristic of the Forum, distinguishing it from many of these efforts, is the inclusivity and breadth of the networks it convenes, including regulatory agencies and patient and community groups, and the emphasis that all stakeholders have an equal voice in discussions. Particularly, the involvement of advocacy and community organizations has been critical to ensuring that the outcomes of the Forum's efforts, including consensus on clinical trial design, benefit those who are affected by the diseases the Forum works on. Moreover, central to the Forum's approach is to be responsive to the needs of the global health community. This is exemplified by the changing and expanding focus of the Forum's work throughout its history and new strategic approaches such as the DAC. As a US‐based organization, the Forum has had, historically, strong connections to regulatory and normative bodies, academic groups, industry, and patient and community organizations in the US, although it has addressed global issues in public health since its inception. For instance, in the early 2000s, the HIV Forum addressed monitoring of long‐term toxicities of HIV treatment from an international perspective.^11^ More recently, several Forum workstreams have adopted the explicit objective of facilitating interactions between stakeholders in different regions globally. The HIV Forum's work on HIV prevention aims to provide a forum of exchange for regulatory authorities and other stakeholders in the US, Europe, and Africa. Similarly, the HBV Forum has initiated a working group on conducting clinical trials in the African region. These efforts have significantly strengthened the Forum's network in the African region, particularly with regulatory authorities, at a time of significant change in the African regulatory landscape, with a drive toward regulatory harmonization under the African Medicines Agency (AMA). A global stakeholder base ensures that the Forum addresses current scientific, regulatory, and policy issues that concern the communities that could benefit most from new pharmaceutical products. This responsiveness will be critical to ensure that the Forum's model will stay relevant and continue to support collaboration in global health.

## FUNDING INFORMATION

No specific funding was received for this work. The Forum for Collaborative Research is supported by project funding from the Bill & Melinda Gates Foundation [INV‐045445] and unrestricted grants from the pharmaceutical industry. This article reports on work conducted by the Forum for Collaborative Research since 1997, and the included references contain further information on funding received for specific projects.

## CONFLICT OF INTEREST STATEMENT

The authors declared no competing interests for this work.
